# Supplementary material for: Data-driven long-term glycaemic control trajectories and their associated health and economic outcomes in Finnish patients with incident type 2 diabetes
Source: PLoS One. 2022 Jun 1;17(6):e0269245. doi: 10.1371/journal.pone.0269245 (PMC9159579; doi:10.1371/journal.pone.0269245)
Supplement: S1 Table — (PDF) [file pone.0269245.s001.pdf]

S1 Table. Definitions of micro- and macrovascular complications and numbers of outcome events by type of analysis (first event occurrence after type 2 diabetes diagnosis).

	ICD-10 code[1] or NOMESCO classification of surgical procedures code[2]	No. of events in analyses of microvascular complications	No. of events in analyses of macrovascular complications	No. of events in analyses of micro- and macrovascular complications
**Microvascular complications**		**125**		**105**
**Eye complications**		**16**		**15**
Retinopathy or other diabetic eye complication	E11.3, H28.0, H36 (excluding H36.8), H40.5, H42.0, H43.1, H45.0	14		14
Blindness in one or two eyes	H54	2		1
**Renal complications**		**34**		**27**
Renal insufficiency	E11.2, N08.3, N18	33		27
End-stage renal disease (with or without dialysis)	Z49, Z94.0	1		0
**Neuropathic complications**		**75**		**63**
Diabetic neuropathy, Charcot foot or other diabetic foot complication	E11.4, E11.5, E11.6, G59.0, G63.2, G73.0, G99.0, I70.2, I73.9, I79.2, L97, M14.2, M14.6, N48.4	75		63
Amputation of lower extremities	NOMESCO: NFQ10, NFQ20, NGQ10, NGQ20, NHQ10, NHQ20, NFQ48, NGQ48, NHQ30, NHQ40, NHQ60	0		0
**Macrovascular complications**			**237**	**213**
**Cardiovascular complications**			**211**	**193**
Angina pectoris	I20		11	9
Chronic heart failure	I11.0, I13.0, I13.2, I50		65	59
Incident myocardial infarction/cardiac arrest	I21, I46		20	18

Other ischemic heart disease	I22–I25		115	107
**Cerebrovascular complications**			**26**	**20**
Ischemic stroke	I63–I66 (excluding I63.6)		26	20

[1]WHO. International Statistical Classification of Diseases and Related Health Problems 10th revision. Available at: http://apps.who.int/classifications/icd10/browse/2010/en. Accessed 10 Oct 2019.

[2]Nordic Medico-Statistical Committee. NOMESCO Classification of Surgical Procedures (NCSP), version 1.15. Available at: https://norden.diva-portal.org/smash/get/diva2:970547/FULLTEXT01.pdf. Accessed 8 Jan 2019.
