# Supplementary material for: Data-driven long-term glycaemic control trajectories and their associated health and economic outcomes in Finnish patients with incident type 2 diabetes
Source: PLoS One. 2022 Jun 1;17(6):e0269245. doi: 10.1371/journal.pone.0269245 (PMC9159579; doi:10.1371/journal.pone.0269245)
Supplement: S2 Table — (PDF) [file pone.0269245.s002.pdf]

S2 Table. Definitions of concordant and discordant diseases at baseline.

**Concordant, T2D-coexisting diseases**	**ICD-10 code[1]**
Hypertension	I10
Coronary heart disease	I20–I25
Atrial fibrillation	I48
Heart failure	I50, I11.0, I13.0, I13.2
Peripheral arterial diseases	I70.2, I73.9
Stroke (incl. Sah)	I60, I61, I63 (excluding I63.6), I64
Chronic kidney disease	N18, N19
Neuropathies	G59, G63, G73, G99
Blindness	H54
Diabetes complications	E11.2–E11.8 sublevels
**Discordant diseases**	
Cancers	C00–C43, C45–C97
Asthma	J45, J46
Gout	M10
Glaucoma	H40–H42
Depression	F32, F33
Dementia	F00–F03, G30
Mental diseases	F20–F48
Chronic obstructive pulmonary disease	J43–J44
Rheumatoid and other arthritis	M05–M13, M32, M33, M45
Osteoporosis	M80–M85
Neuromuscular diseases	G70–G72
Liver diseases excluding cancers	K70–K77

[1]WHO. International Statistical Classification of Diseases and Related Health Problems 10th revision. Available at: http://apps.who.int/classifications/icd10/browse/2010/en. Accessed 10 Oct 2019.
