# Supplementary material for: Data-driven long-term glycaemic control trajectories and their associated health and economic outcomes in Finnish patients with incident type 2 diabetes
Source: PLoS One. 2022 Jun 1;17(6):e0269245. doi: 10.1371/journal.pone.0269245 (PMC9159579; doi:10.1371/journal.pone.0269245)
Supplement: S3 Table — (PDF) [file pone.0269245.s003.pdf]

S3 Table. Unadjusted latent class growth analyses for HbA1c values measured annually since T2D diagnosis among patients diagnosed in 2011 or 2012 (n=1540).

**Model**	**Log-likelihood**	**N of param**	**BIC**	**Entropy**	**LMR-LRT *P***	**BLRT *P***	***Min. APP***	***Class 1***	***Class 2***	***Class 3***	***Class 4***	***Class 5***
**Linear**												
**1-class**	-27 186.3	10	54 446.1	NA	NA	NA	NA	100.0				
**2-class**	-25 981.6	13	52 058.7	0.925	0.036	<0.001	0.957	87.9	12.1			
**3-class**	-25 621.2	16	51 359.8	0.816	0.040	<0.001	0.874	71.2	21.8	7.1		
**4-class**	-25 339.6	19	50 818.7	0.840	0.043	<0.001	0.856	68.1	23.4	5.8	2.7	
**5-class**	-25 197.7	22	50 556.9	0.849	0.193	<0.001	0.847	66.2	24.4	4.5	3.9	1.1
**Quadratic**												
**1-class**	-27 169.5	11	54 419.7	NA	NA	NA	NA	100.0				
**2-class**	-25 964.1	15	52 038.2	0.928	0.041	<0.001	0.952	87.8	12.2			
**3-class**	-25 603.6	19	51 346.6	0.816	0.422	<0.001	0.865	70.6	22.3	7.0		
**4-class**	-25 259.0	23	50 686.7	0.845	0.216	<0.001	0.857	68.2	23.7	5.7	2.4	
**5-class**	-25 054.2	27	50 306.5	0.849	0.261	<0.001	0.845	65.6	24.7	6.3	2.4	0.9
**Cubic**												
**1-class**	-27 158.9	12	54 405.9	NA	NA	NA	NA	100.0				
**2-class**	-25 933.7	17	51 992.3	0.930	0.051	<0.001	0.949	87.6	12.4			
**3-class**	-25 558.4	22	51 278.4	0.822	0.406	<0.001	0.870	71.2	21.8	7.0		
**4-class**	-25 211.0	27	50 620.1	0.844	0.343	<0.001	0.855	68.6	22.1	6.1	3.2	
**5-class**	-24 972.6	32	50 180.0	0.860	0.217	<0.001	0.851	66.6	23.5	5.9	3.1	0.8

Abbreviations: APP, average posterior probability; BIC, Bayesian information criteria; LMR-LRT, Lo-Mendell-Rubin likelihood ratio test; NA, not available.
