# Supplementary material for: Data-driven long-term glycaemic control trajectories and their associated health and economic outcomes in Finnish patients with incident type 2 diabetes
Source: PLoS One. 2022 Jun 1;17(6):e0269245. doi: 10.1371/journal.pone.0269245 (PMC9159579; doi:10.1371/journal.pone.0269245)
Supplement: S4 Table — (PDF) [file pone.0269245.s004.pdf]

S4 Table. Antidiabetic medication use (%) by the trajectories after the T2D diagnosis.

	1st year	2nd year	3rd year	4th year	5th year	6th year	7th year
**Metformin**							
Stable, adequate	42.2	44.3	44.7	44.9	46.2	47.3	48.2
Slowly deteriorating	62.9	63.1	65.3	66.8	68.6	67.2	68.1
Rapidly deteriorating	67.4	58.7	59.3	56.0	55.0	56.2	58.6
Late diagnosed	61.7	65.2	65.9	65.1	64.1	65.7	56.3
**Sulfonylureas**							
Stable, adequate	0.5	0.4	0.4	0.3	0.3	0.3	0.3
Slowly deteriorating	2.1	2.4	1.2	1.2	1.6	1.4	1.4
Rapidly deteriorating	1.1	1.1	1.1	1.2	1.3	2.7	0.0
Late diagnosed	4.3	2.2	0	2.3	2.6	2.9	3.1
**Combinations of oral BG lowering drugs**							
Stable, adequate	1.2	1.1	1.5	1.7	1.8	2.0	2.2
Slowly deteriorating	1.2	1.5	3.3	5.0	7.1	8.4	10.6
Rapidly deteriorating	3.2	5.4	7.7	9.5	7.5	9.6	14.3
Late diagnosed	6.4	6.5	9.1	9.3	10.3	20.0	21.9
**Glitazones**							
Stable, adequate	0.0	0.1	0.1	0.0	0.0	0.0	0.0
Slowly deteriorating	0.0	0.0	0.3	0.3	0.3	0.3	0.4
Rapidly deteriorating	1.1	1.1	1.1	2.4	1.3	1.4	2.9
Late diagnosed	0.0	0.0	0.0	0.0	0.0	0.0	0.0
**DPP-4 inhibitors**							
Stable, adequate	4.3	5.0	5.9	6.7	7.9	8.8	9.3
Slowly deteriorating	11.8	17.4	22.4	27.0	33.3	34.8	39.0
Rapidly deteriorating	34.7	28.3	30.8	39.3	48.8	47.9	41.4
Late diagnosed	17.0	32.6	36.4	30.2	38.5	40.0	46.9
**Glinides**							
Stable, adequate	0.2	0.2	0.2	0.6	0.3	0.0	0.0
Slowly deteriorating	0.3	0.3	0.6	0.9	3.5	1.0	1.1
Rapidly deteriorating	0.0	1.1	3.3	3.6	6.3	1.4	0.0
Late diagnosed	2.1	6.5	9.1	7.0	7.7	2.9	3.1
**GLP-1 analogues**							
Stable, adequate	0.0	0.0	0.0	0.1	0.3	0.3	0.5
Slowly deteriorating	0.0	0.0	0.0	0.0	0.3	2.0	4.3
Rapidly deteriorating	0.0	0.0	0.0	1.2	7.5	8.2	11.4
Late diagnosed	0.0	0.0	0.0	0	7.7	8.6	3.1
**SGLT2 inhibitors**							
Stable, adequate	0.0	0.0	0.0	0.1	1.0	2.0	3.4
Slowly deteriorating	0.0	0.0	0.0	0.9	7.1	17.9	25.9
Rapidly deteriorating	0.0	0.0	0.0	0	8.8	35.6	34.3
Late diagnosed	0.0	0.0	0.0	0	5.1	11.4	15.6
**Insulin**							
Stable, adequate	1.1	1.1	1.3	0.8	1.3	1.2	1.2
Slowly deteriorating	5.0	5.6	6.9	8.1	9.0	10.5	11.7
Rapidly deteriorating	20.0	22.8	24.2	29.8	37.5	43.8	48.6
Late diagnosed	48.9	43.5	43.2	48.8	46.2	37.1	37.5

Abbreviations: BG, blood glucose; DPP-4, dipeptidyl peptidase 4; GLP-1, glucagon-like peptide-1; SGLT2, sodium/glucose cotransporter 2; T2D, type 2 diabetes.
