# Supplementary material for: Data-driven long-term glycaemic control trajectories and their associated health and economic outcomes in Finnish patients with incident type 2 diabetes
Source: PLoS One. 2022 Jun 1;17(6):e0269245. doi: 10.1371/journal.pone.0269245 (PMC9159579; doi:10.1371/journal.pone.0269245)
Supplement: S5 Table — (PDF) [file pone.0269245.s005.pdf]

S5 Table. Associations between the estimated HbA1c trajectories and incidence of deaths and diabetes complications.

	Events (%)	Events/100 person-years (95% CI)	Unadjusted HR (95% CI)	Age- and sex-adjusted HR (95% CI)	Fully adjusted[a] HR (95% CI)
**All-cause death**					
Stable, adequate glycaemic control	128 (12.1)	2.20 (1.85–2.61)	1.00 (reference)	1.00 (reference)	1.00 (reference)
Slowly deteriorating glycaemic control	61 (17.9)	3.16 (2.44–4.03)	1.42 (1.04–1.92)	1.30 (0.96–1.77)	1.24 (0.91–1.69)
Rapidly deteriorating glycaemic control	24 (25.3)	4.70 (3.08–6.89)	2.14 (1.38–3.31)	2.58 (1.67–3.99)	2.49 (1.61–3.87)
Late diagnosed patients	13 (27.7)	5.21 (2.89–8.67)	2.38 (1.34–4.20)	3.33 (1.88–5.90)	2.86 (1.60–5.12)
**Micro- and macrovascular complications**					
Stable, adequate glycaemic control	189 (17.9)	3.49 (3.01–4.01)	1.00 (reference)	1.00 (reference)	1.00 (reference)
Slowly deteriorating glycaemic control	88 (25.9)	5.07 (4.08–6.22)	1.44 (1.11–1.85)	1.37 (1.07–1.77)	1.39 (1.08–1.79)
Rapidly deteriorating glycaemic control	26 (27.4)	5.50 (3.61–8.07)	1.73 (1.15–2.61)	2.12 (1.41–3.20)	2.28 (1.51–3.46)
Late diagnosed patients	15 (31.9)	7.32 (4.25–11.8)	2.08 (1.23–3.51)	2.68 (1.58–4.55)	3.35 (1.96–5.72)
**Microvascular complications**					
Stable, adequate glycaemic control	62 (5.9)	1.06 (0.81–1.35)	1.00 (reference)	1.00 (reference)	1.00 (reference)
Slowly deteriorating glycaemic control	39 (11.5)	2.09 (1.50–2.84)	1.94 (1.30–2.89)	1.91 (1.28–2.84)	2.00 (1.34–2.99)
Rapidly deteriorating glycaemic control	16 (16.8)	3.04 (1.73–4.98)	3.23 (1.86–5.59)	3.60 (2.07–6.26)	3.68 (2.11–6.43)
Late diagnosed patients	8 (17.0)	3.55 (1.65–6.75)	3.20 (1.53–6.68)	3.58 (1.71–7.50)	4.46 (2.10–9.47)
**Macrovascular complications**					
Stable, adequate glycaemic control	147 (13.9)	2.69 (2.28–3.15)	1.00 (reference)	1.00 (reference)	1.00 (reference)
Slowly deteriorating glycaemic control	63 (18.5)	3.49 (2.70–4.43)	1.27 (0.95–1.71)	1.19 (0.88–1.60)	1.20 (0.89–1.62)
Rapidly deteriorating glycaemic control	16 (16.8)	3.36 (1.99–5.33)	1.25 (0.74–2.09)	1.53 (0.91–2.57)	1.62 (0.97–2.72)
Late diagnosed patients	11 (23.4)	4.95 (2.61–8.61)	1.85 (1.00–3.41)	2.56 (1.38–4.73)	3.18 (1.71–5.94)

Abbreviations: CI, confidence interval; HR, hazard ratio.

[a]Adjusted for age, sex, concordant diseases, discordant diseases, microvascular and macrovascular comorbidities at baseline.
