# Supplementary material for: Data-driven long-term glycaemic control trajectories and their associated health and economic outcomes in Finnish patients with incident type 2 diabetes
Source: PLoS One. 2022 Jun 1;17(6):e0269245. doi: 10.1371/journal.pone.0269245 (PMC9159579; doi:10.1371/journal.pone.0269245)
Supplement: S6 Table — All the costs are adjusted for censoring due to death utilizing Kaplan-Meier sample average estimator. (PDF) [file pone.0269245.s006.pdf]

S6 Table. Average accumulated 4-year per-person healthcare costs (euros, €) by HbA1c trajectories in 2014–2017. All the costs are adjusted for censoring due to death utilizing Kaplan-Meier sample average estimator.

**Trajectory**	**Stable, adequate glycaemic control**	**Slowly deteriorating glycaemic control**	**Rapidly deteriorating glycaemic control**	**Late diagnosed patients**
**n (%), alive on Jan 1, 2014**	1032 (68.7)	335 (22.1)	90 (6.2)	44 (3.1)
**Accumulated total costs**	15 341	19 229	16 284	17 987
1. Accumulated social- and healthcare costs	12 124	15 312	11 952	13 358
**i) Primary healthcare costs**	3006	3610	3457	2465
-Outpatient visits	1659	1713	1724	1022
-Inpatient visits	1347	1897	1732	1442
**ii) Specialized healthcare costs**	4543	5323	4589	3005
-Outpatient visits	1920	1820	1476	881
-Inpatient visits	2622	3503	3113	2124
**iii) Nursing home care costs**	1200	2946	782	437
**iv) Home care costs**	3376	3433	3124	7451
2. Accumulated medication costs	3216	3917	4333	4629
**i) Accumulated diabetes medication costs**	363	1163	2433	2396
