# Supplementary material for: Data-driven long-term glycaemic control trajectories and their associated health and economic outcomes in Finnish patients with incident type 2 diabetes
Source: PLoS One. 2022 Jun 1;17(6):e0269245. doi: 10.1371/journal.pone.0269245 (PMC9159579; doi:10.1371/journal.pone.0269245)
Supplement: Attachment — Submitted filename: Response to reviewers.docx [file pone.0269245.s007.docx]

Reviewers' comments:

Reviewer's Responses to Questions

**Comments to the Author**

1. Is the manuscript technically sound, and do the data support the conclusions?

The manuscript must describe a technically sound piece of scientific research with data that supports the conclusions. Experiments must have been conducted rigorously, with appropriate controls, replication, and sample sizes. The conclusions must be drawn appropriately based on the data presented.

Reviewer #1: Yes

Reviewer #2: Yes

2. Has the statistical analysis been performed appropriately and rigorously?

Reviewer #1: Yes

Reviewer #2: I Don't Know

3. Have the authors made all data underlying the findings in their manuscript fully available?

The [PLOS Data policy](https://eur03.safelinks.protection.outlook.com/?url=http%3A%2F%2Fwww.plosone.org%2Fstatic%2Fpolicies.action%23sharing&data=04%7C01%7C%7C37b85a04b3104e61bed408da1404a26e%7C87879f2e73044bf2baf263e7f83f3c34%7C0%7C0%7C637844308414265479%7CUnknown%7CTWFpbGZsb3d8eyJWIjoiMC4wLjAwMDAiLCJQIjoiV2luMzIiLCJBTiI6Ik1haWwiLCJXVCI6Mn0%3D%7C3000&sdata=%2BxkKTTQX3NAws3S7bpTlKM9R%2BIVMTo0qbmJkqmOOAKM%3D&reserved=0) requires authors to make all data underlying the findings described in their manuscript fully available without restriction, with rare exception (please refer to the Data Availability Statement in the manuscript PDF file). The data should be provided as part of the manuscript or its supporting information, or deposited to a public repository. For example, in addition to summary statistics, the data points behind means, medians and variance measures should be available. If there are restrictions on publicly sharing data—e.g. participant privacy or use of data from a third party—those must be specified.

Reviewer #1: Yes

Reviewer #2: Yes

4. Is the manuscript presented in an intelligible fashion and written in standard English?

PLOS ONE does not copyedit accepted manuscripts, so the language in submitted articles must be clear, correct, and unambiguous. Any typographical or grammatical errors should be corrected at revision, so please note any specific errors here.

Reviewer #1: Yes

Reviewer #2: Yes

5. Review Comments to the Author

Please use the space provided to explain your answers to the questions above. You may also include additional comments for the author, including concerns about dual publication, research ethics, or publication ethics. (Please upload your review as an attachment if it exceeds 20,000 characters)

**Reviewer #1:** a useful work which confirms already accepted knowledge regarding the nature and natural history of diabetes, would have been add on to currently known data if there was any stratifications of the drug used and if there was any impact of the class of drugs especially new classes with presumed additive benefits on cardiovascular outcomes rather than just focusing on the progression of the disease

**Our response**: *We thank the Reviewer for the comments. However, we would prefer not to conduct additional stratified analyses. First, diabetes medication use changes over time as is seen from figure 2 and table S4, which also show how medication use modifies HbA1c trajectories. Modeling such a time-varying factor that is also associated with HbA1c would be challenging in this study and we feel that stratified analyses would require different study design. Second, we have submitted another study where we have examined changes in diabetes medication use and its association with treatment outcomes.*

**Reviewer #2:** Piia Lavikainen et al have applied a clustering strategy to study patients with newly diagnosed type 2 diabetes and examined the association of these groups with heath outcomes including all-cause death and incident micro- and macrovascular complications.
4 groups were identified using this latent-class growth analysis clustering approach : “Stable, adequate” (large majority, 2/3 of the study population), “Slowly deteriorating” (second largest, one fourth of the population) and 2 smal lgroups “Rapidly deteriorating” glycaemic control (6.2%) and “Late diagnosed” patients (3.4%). Most of the interest is focused on these 2 last groups.
The paper is interesting but an effort should be made to facilitate direct access to salient clinical results

General comment
I see A1c not only as a biomarker of treatment efficacy but also as a possible biomarker indicative of severity of the disease as used in Ahlquist intial clustering work (Lancet DE). In addition the response of A1c cannot be seen as a universal response to drugs since diabetes drugs are limited to 8 large classes in patients with T2D (SU/glinides ; TZDs; Metformin; DPP4-Is ; SGLT2-Is; AG-Is; GLP1RA; insulin) although AG-Is do not appear in the current paper.

**Our response:** *We have added to the Discussion section on baseline clustering conducted by Ahlqvist et al. as follows: “To note, also clustering based on patient characteristics (age, HbA1c, BMI, homeostasis model assessment (HOMA2) estimates of β-cell function and insulin resistance) at the time of diagnosis in general diabetic population has been conducted[28] instead of longitudinal clustering based on HbA1c solely.”*

*We have also discussed about the HbA1c being possible indicator of disease severity as follows: “HbA1c may not reflect only treatment response but also the underlying severity of the disease[28]. Late diagnosed patients used most often insulin reflecting more severe disease, seen also as higher incidence of complications during the 7-year follow-up despite of reaching good glycaemic control 2–3 years after the diagnosis.”*

*AG-Is are not included in the paper as they are not used in Finland. AG-Is are not mentioned in the treatment guidelines of type 2 diabetes in Finland, and there seems to be no AG-I products on the market at the moment.*

Table 1
The authors should present data on eGFR and not only serum creatinine values.
Do the authors have access to Fasting plasma glucose at diagnosis ? it would help to confront FPG and A1c.

**Our response:** *We thank the Reviewer for the suggestion and have added eGFR and FPG to the table 1. FPG confronts the observed HbA1c values at baseline. Serum creatinine values were converted to eGFR* *utilizing the CKD-EPI (Chronic Kidney Disease Epidemiology Collaboration) equation (Levey et al. Ann Intern Med 2009;150:604-612) that accounts for age and gender.*

Table 3 needs to be more directly self-comprehensive as the amount should correspond to yearly expenses (please clarify this)

**Our response:** *We thank the Reviewer for commenting on this. We have clarified that the table presents average accumulated 4-year per-person costs over 2014-2017 for the stable cluster and additional costs in other clusters compared with the stable one. We would prefer presenting the accumulated costs along the HbA1c trajectories rather than presenting average yearly costs although they are often reported in cost-effectiveness analyses.*

I am wondering how to adjust on baseline characteristics which are different between the 4 selected groups, particularly age and sex, regarding the effect of each group on complications. This would be appreciated.

**Our response:** *We thank the Reviewer for noticing this. Individual level Cox regression models were used to examine adjusted associations of the 4 groups with complications. The models were adjusted for age, sex, concordant and discordant diseases, and microvascular and macrovascular comorbidities of a patient at baseline. Unfortunately, we had not mentioned this in the paragraph of Statistical analyses. Therefore, we have refined the text as follows: “Models were adjusted for age, sex, concordant and discordant diseases, and microvascular and macrovascular comorbidities at baseline.”*

The discussion is interesting and could however be shortened.

**Our response:** *We have shortened the discussion by removing, for example, detailed description of HbA1c clusters identified in previous studies.*

6. PLOS authors have the option to publish the peer review history of their article ([what does this mean?](https://eur03.safelinks.protection.outlook.com/?url=https%3A%2F%2Fjournals.plos.org%2Fplosone%2Fs%2Feditorial-and-peer-review-process%23loc-peer-review-history&data=04%7C01%7C%7C37b85a04b3104e61bed408da1404a26e%7C87879f2e73044bf2baf263e7f83f3c34%7C0%7C0%7C637844308414265479%7CUnknown%7CTWFpbGZsb3d8eyJWIjoiMC4wLjAwMDAiLCJQIjoiV2luMzIiLCJBTiI6Ik1haWwiLCJXVCI6Mn0%3D%7C3000&sdata=%2BX6prEB0dOXQ7duNQHtl5PE6fjJZZgTlV0Zb745Qtmo%3D&reserved=0)). If published, this will include your full peer review and any attached files.


If you choose “no”, your identity will remain anonymous but your review may still be made public.


**Do you want your identity to be public for this peer review?** For information about this choice, including consent withdrawal, please see our [Privacy Policy](https://eur03.safelinks.protection.outlook.com/?url=https%3A%2F%2Fwww.plos.org%2Fprivacy-policy&data=04%7C01%7C%7C37b85a04b3104e61bed408da1404a26e%7C87879f2e73044bf2baf263e7f83f3c34%7C0%7C0%7C637844308414265479%7CUnknown%7CTWFpbGZsb3d8eyJWIjoiMC4wLjAwMDAiLCJQIjoiV2luMzIiLCJBTiI6Ik1haWwiLCJXVCI6Mn0%3D%7C3000&sdata=l883K7RiJJjC%2Fc0OZFZSwCkc73A5xT1QNUPl4TRQShU%3D&reserved=0).

Reviewer #1: No

Reviewer #2: No
